# Machine learning detection of Atrial Fibrillation using wearable technology

**DOI:** 10.1371/journal.pone.0227401

**Published:** 2020-01-24

**Authors:** Mark Lown, Michael Brown, Chloë Brown, Arthur M. Yue, Benoy N. Shah, Simon J. Corbett, George Lewith, Beth Stuart, Michael Moore, Paul Little

**Affiliations:** 1 Primary Care & Population Sciences, Faculty of Medicine, University of Southampton, Southampton, England; 2 Leonardo MW Ltd, Southampton, England; 3 Cardiology and Electrophysiology, Southampton General Hospital, Southampton, England; University of Minnesota, UNITED STATES

## Abstract

**Background:**

Atrial Fibrillation is the most common arrhythmia worldwide with a global age adjusted prevalence of 0.5% in 2010. Anticoagulation treatment using warfarin or direct oral anticoagulants is effective in reducing the risk of AF-related stroke by approximately two-thirds and can provide a 10% reduction in overall mortality. There has been increased interest in detecting AF due to its increased incidence and the possibility to prevent AF-related strokes. Inexpensive consumer devices which measure the ECG may have the potential to accurately detect AF but do not generally incorporate diagnostic algorithms. Machine learning algorithms have the potential to improve patient outcomes particularly where diagnoses are made from large volumes or complex patterns of data such as in AF.

**Methods:**

We designed a novel AF detection algorithm using a de-correlated Lorenz plot of 60 consecutive RR intervals. In order to reduce the volume of data, the resulting images were compressed using a wavelet transformation (JPEG200 algorithm) and the compressed images were used as input data to a Support Vector Machine (SVM) classifier. We used the Massachusetts Institute of Technology (MIT)—Beth Israel Hospital (BIH) Atrial Fibrillation database and the MIT-BIH Arrhythmia database as training data and verified the algorithm performance using RR intervals collected using an inexpensive consumer heart rate monitor device (Polar-H7) in a case-control study.

**Results:**

The SVM algorithm yielded excellent discrimination in the training data with a sensitivity of 99.2% and a specificity of 99.5% for AF. In the validation data, the SVM algorithm correctly identified AF in 79/79 cases; sensitivity 100% (95% CI 95.4%-100%) and non-AF in 328/336 cases; specificity 97.6% (95% CI 95.4%-99.0%).

**Conclusions:**

An inexpensive wearable heart rate monitor and machine learning algorithm can be used to detect AF with very high accuracy and has the capability to transmit ECG data which could be used to confirm AF. It could potentially be used for intermittent screening or continuously for prolonged periods to detect paroxysmal AF. Further work could lead to cost-effective and accurate estimation of AF burden and improved risk stratification in AF.

## Introduction

Atrial Fibrillation is the most common arrhythmia worldwide with a global age adjusted prevalence of 0.5% in 2010.[1] Numerous studies have reported a growing epidemic of AF with an expected doubling of its prevalence by 2030.[1]AF is linked with up to 1/3 of all strokes and as a significant proportion is asymptomatic it can often go undiagnosed.[2] There has been increased interest in detecting AF due to its increased incidence and the possibility to prevent AF-related strokes.[3] Anticoagulation treatment using warfarin or direct oral anticoagulants is effective in reducing the risk of AF-related stroke by approximately two-thirds and can provide a 10% reduction in overall mortality.[4] Screening for AF has been the subject of much recent debate by expert committees.[3]

There has been recent interest in the use of consumer devices for the detection of arrhythmias including AF.[5,6] Wrist-worn heart-rate sensors that measure heart rate using Light Emitting Diodes (LEDs) have become increasingly popular but their accuracy has been questioned and some manufacturers advise the heart rate measurement should be considered an estimate and suggest using a chest heart rate sensor for increased accuracy.[7] LED based wrist sensors have been shown to underestimate heart-rate during AF and thus may be less accurate for detecting AF.[8] Because ECG confirmation is mandated by guidelines for the diagnosis of AF, devices which sense an ECG signal may have the advantage of providing a verifiable ECG trace and would therefore be a preferred screening tool.[9] Although consumer chest heart rate sensors may be less comfortable than wrist based sensors, they are inherently designed to be compact, lightweight, unobtrusive, have long battery lives and should be accurate when used in sub-optimal settings such as during exercise. Consumer devices are now available which transmit ECG data in addition to heart rate enabling accurate AF detection by a cardiologist and automated algorithms.[10]

Consumer devices do not generally have algorithms incorporated for the detection of AF. In terms of diagnostic algorithms, machine learning has the potential to improve patient outcomes particularly where diagnoses are made from large volumes or complex patterns of data. Recently, machine learning algorithms have been developed to detect diabetic retinopathy with high accuracy[11] and to detect breast cancer metastases with a higher accuracy than pathologists.[12] In this research we aimed to investigate if an inexpensive consumer chest heart rate monitor device could be used along with a highly discriminative machine learning algorithm to accurately detect AF.

## Methods

The study complies with the declaration of Helsinki, and the protocol was approved by the London—City & East Research Ethics Committee in June 2016 (ref 16/LO/1173). Informed consent was obtained from all participants (trial registration ISRCTN: 17495003). We developed the algorithm using an online database of RR intervals obtained from Holter recordings containing segments of sinus rhythm, AF and other arrhythmias.[13] We used 60 consecutive intervals to construct a Lorenz plot (a correlated scatter-plot of consecutive RR interval changes vs the previous change (ΔRR[k], ΔRR[k-1]) depicting heart rate variability). The patterns of data in the Lorenz plots for regularly irregular intervals such as bigeminy can distinguished from the more random pattern of AF.[14] We de-correlated the Lorenz plot using the following direct transformation derived from first principles (see S1 Appendix):

$$Y'=\frac{Y+0.5X}{\sqrt{0.75}}$$ (1)

**Fig 1:**
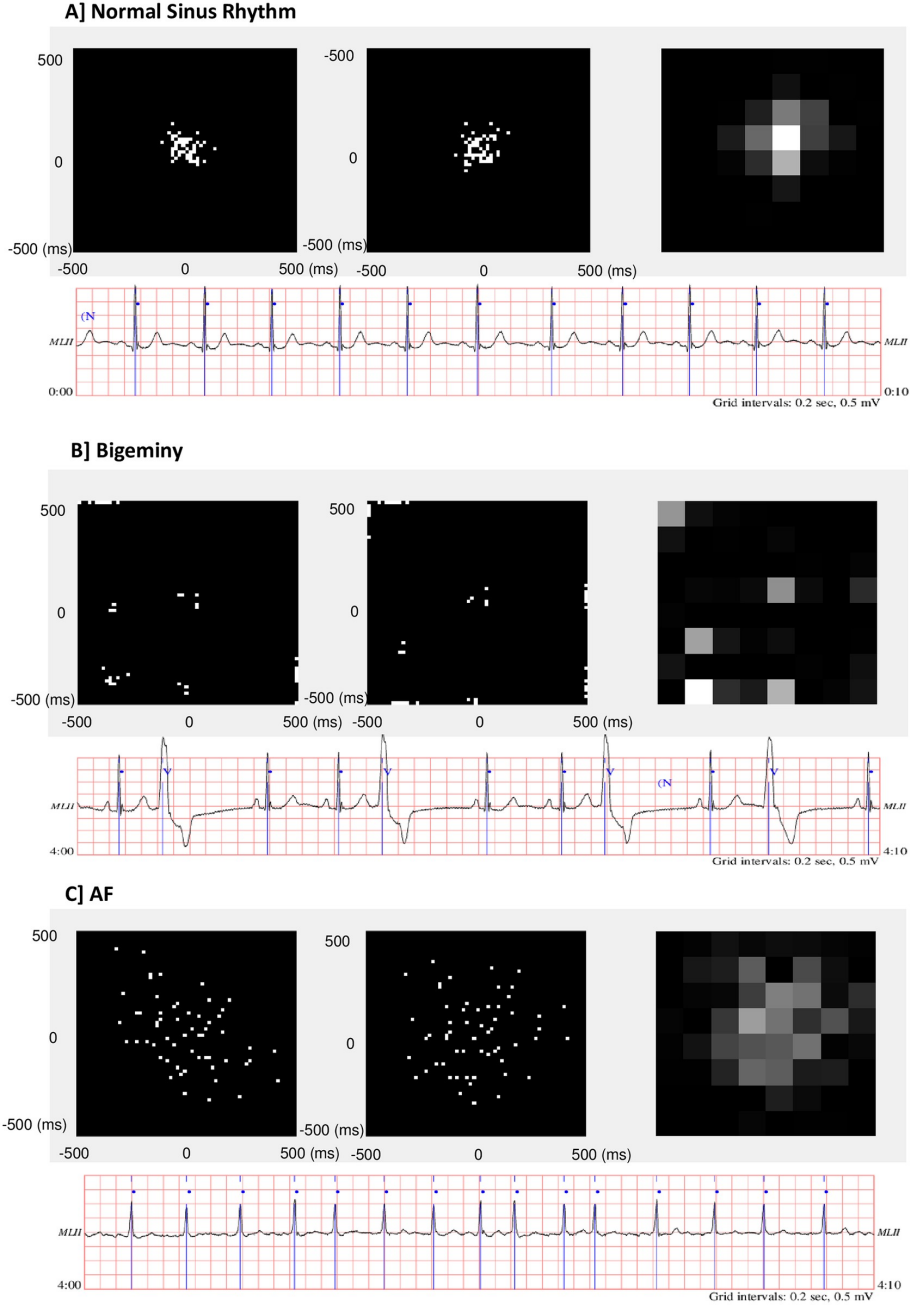
Correlated de-correlated Lorenz plots, compressed images and corresponding ECG data used to train the SVM for 60 interval segments of [A] normal sinus rhythm, [B] bigeminy and [C] AF.

Where X is the difference between the two consecutive, discrete RR intervals and Y is the previous value of X ie. X = RR(k)–RR(k-1) and Y = X(k-1). We then compressed the de-correlated Lorenz plot using a wavelet-transformation (as used in the JPEG 2000 image compression algorithm[15]) to reduce the volume of data (to an 8x8 pixel image) presented to the machine learning algorithm (a Gaussian Support Vector Machine (SVM)) and hence the volume of training data required. Overtraining was avoided using crossfold-validation through the dataset.[16] (K-fold cross validation partitions data into k randomly chosen subsets (or folds) of roughly equal size. One subset is used to validate the model trained using the remaining (k-1) subsets. This process is repeated k times such that each subset is used exactly once for validation. We used 5-fold cross validation and the data was split randomly across records in the entire training dataset). This process is depicted in Fig 1 which depicts the original Lorenz plots, de-correlated Lorenz plots and subsequent compressed images used to train the SVM for 60 interval segments of normal sinus rhythm, bigeminy and AF. 60 consecutive RR intervals were used for training and in the validation data.

We trained the SVM using the Massachusetts Institute of Technology (MIT)—Beth Israel Hospital (BIH) Atrial Fibrillation database (comprising approximately 250 hours from 25 subjects; approximately 95 hours of AF (319 episodes), 2 hours of atrial flutter (40 episodes) and 163 hours of other rhythms) and the MIT-BIH Arrhythmia database (comprising approximately 24 hours of data from 47 subjects including complex ventricular, junctional, and supraventricular arrhythmias). We used the RR intervals and rhythms annotated in the database and did not employ a separate QRS detection algorithm. The MITBIH-AF database yielded 7,744 consecutive 60 beat sequences of AF, and 10,467 non-AF; the MIT-BIH Arrhythmia yielded 134 AF and 1404 non-AF consecutive 60-beat sequences.

We validated the algorithm using data from a case-control study of 415 participants aged > 65yrs (79 with AF at the study visit and 336 without) attending 3 general practice surgeries in Hampshire, UK for a single screening visit.[17] Following informed consent, 60 consecutive R-R intervals were obtained from participants at rest using the Polar H7 (PH7) heart rate monitor (via Bluetooth) and an iPad by study nurses. The participants each had a 12-Lead ECG, which were interpreted independently by 2 cardiologists, with a 3^rd^ cardiologist adjudicating disagreements. The PH7 is an inexpensive device (cost price £26) and has been shown to measure RR intervals accurately.[18]

## Results

### Training data

In the training data, the SVM algorithm yielded excellent discrimination with a sensitivity of 99.2% and a specificity of 99.5% for AF.

#### Validation / Trial data

**Fig 2:**
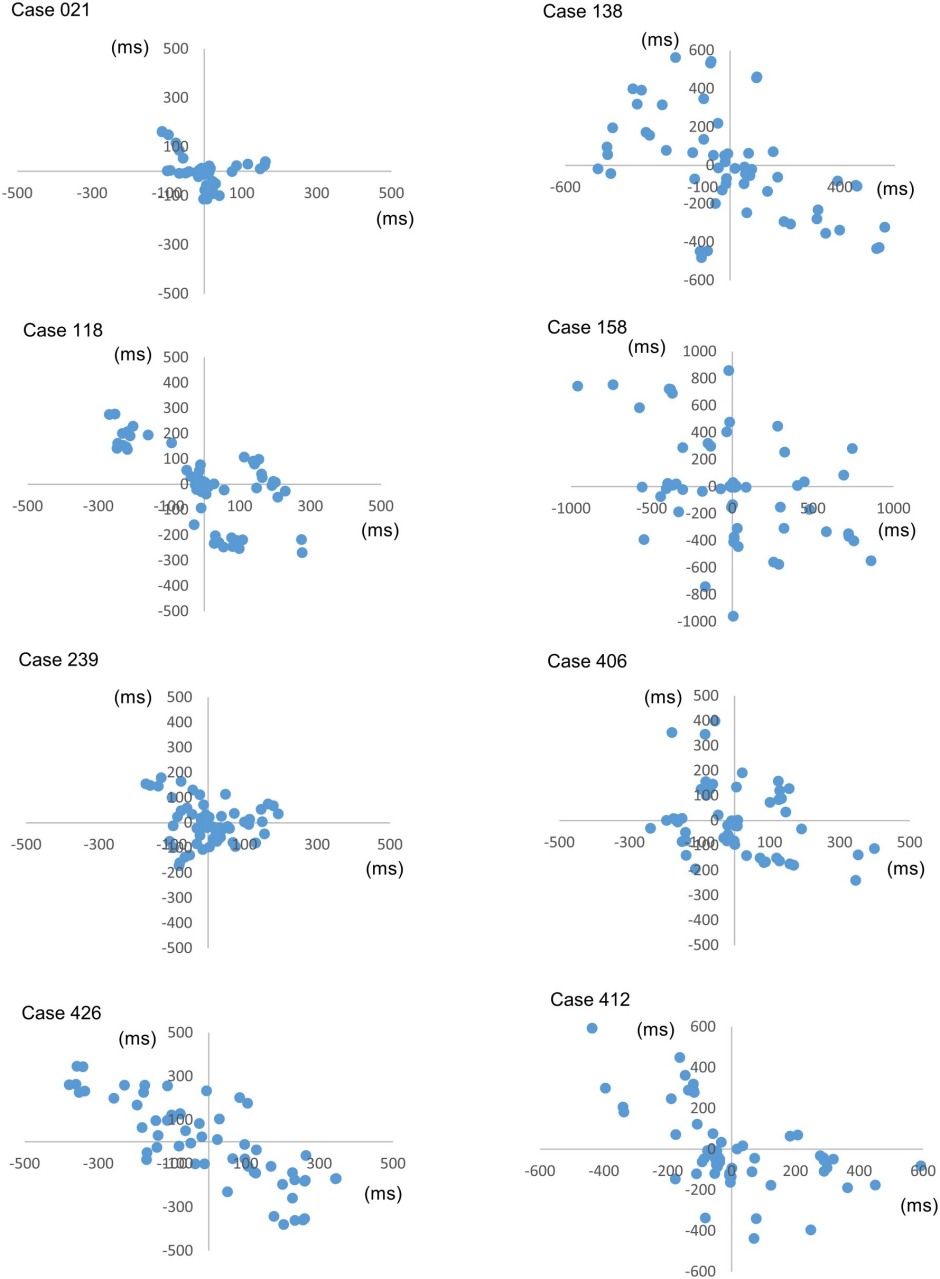
Correlated Lorenz plots for the false positive cases.

The SVM algorithm correctly identified AF in 79/79 cases; sensitivity 100% (95% CI 95.4%-100%) and non-AF in 328/336 cases; specificity 97.6% (95% CI 95.4%-99.0%). Several control cases had non-AF arrhythmias including heart block, bigeminy and sinus arrhythmia[19]. Fig 2 shows Lorenz plots (standard / correlated) for the incorrectly classified (false positive) cases.

## Discussion of results

The SVM algorithm demonstrated excellent discrimination with sensitivity and specificity both exceeding 99% for the training data set. This compares favourably with other algorithms results using the same databases in the literature.[20–22] Furthermore, we have validated the algorithm performance with clinical data obtained using an in expensive consumer device and achieved excellent discrimination with sensitivity and specificity both within 2% of the training data set results. High performing algorithms such as the SVM could greatly reduce clinical workload in terms of clinician confirmation of AF in positive cases. Visual inspection of the Lorenz plots of the false positive cases indicates several cases with clustering of RR intervals (case 021 and 118) which is not suggestive of atrial fibrillation and thus algorithm modifications or further training could possibly lead to improvements in performance.

The accuracy of the Polar-H7 and SVM algorithm also compares favourably with the accuracy of commercial devices designed and approved to detect AF.[23,24] The accuracy of the consumer device and algorithm was comparable to a cardiologist’s interpretation of single-lead ECGs.[25] Although it is not currently implemented commercially, a variant of the Polar-H7 device can transmit single lead ECG data which could be used to confirm the AF diagnosis. A newly available similar device that also provides a rhythm strip has been used clinically to detect AF and the ECG strip was sufficient for reliable confirmation of AF compared with a Holter monitor.[10] Smartphones are becoming more widely used among the elderly and in developing countries and in conjunction with inexpensive wearable technology, present an opportunity for cost-effective screening for AF. If these products are to be used in future for the diagnosis of AF, they would need to be registered as medical devices with either FDA approval (US) or a CE mark (EU).

Current guidelines make identical recommendations for anticoagulation regardless of AF pattern or burden (defined as the amount of time spent in AF). Reviews of recent evidence however suggests that higher AF burden is associated with higher risk of stroke[26]. Developments in monitoring technologies and algorithms will likely change the landscape of long-term AF monitoring and could allow better definition of the significance of changes in AF burden over time and to improved risk stratification and potentially improved patient outcomes. Inexpensive devices such as the Polar-H7 and Suunto Movesense[10] together with further algorithm development and implementation could lead to cost-effective methods for prolonged and continuous monitoring for AF detection. In addition, they could also potentially be used where clinically appropriate for the investigation of palpitations and syncope and the detection of other arrhythmias. The SVMs utility could potentially be used to classify patterns generated by other arrhythmias from the Lorenz plots which would potentially be an advantage over methods employing statistical analysis. This should be considered for future research.

## Conclusions

An inexpensive wearable heart rate monitor and machine learning algorithm can be used to detect AF with very high accuracy and has the capability to transmit ECG data which could be used to confirm AF. It could potentially be used for intermittent screening or continuously for prolonged periods to detect paroxysmal AF. Further work could lead to cost-effective and accurate estimation of AF burden and improved risk stratification in AF.

## Supporting information

S1 AFPlosOne (XLSX)

S1 Polar data (7Z)

S1 Appendix (DOCX)
