# Supplementary material for: Machine learning detection of Atrial Fibrillation using wearable technology
Source: PLoS One. 2020 Jan 24;15(1):e0227401. doi: 10.1371/journal.pone.0227401 (PMC6980577; doi:10.1371/journal.pone.0227401)
Supplement: S1 Appendix — (DOCX) [file pone.0227401.s003.docx]

**Decorrelating the standard Lorenz Plot**

If an RR time sequence is denoted by *x(t),* then the standard Lorenz plot is a 2D graph of *X=[x(t)-x(t-1)]* versus *Y=[x(t-1)-x(t-2)].* It is assumed that a Lorenz plot for a typical NSR rhythm should look like a 2D Normal (bivariate Gaussian) distribution, i.e. a symmetrical “Mexican hat” pattern should be visually apparent, with an equal distribution of points in each quadrant. However the coordinates of the standard Lorenz plot (*X, Y*) are correlated, since they both contain the term *x(t-1).* Thus, even if the RR sequence*, x(t),* is Normally distributed, the Lorenz plot will show a correlation, potentially leading to misclassification of “normal” behaviour as skewed, and thus “abnormal”. Inspection of a standard Lorenz plot with enough RR time sequence data will reveal this correlation.

The correlation effect inherent in a standard Lorenz plot is demonstrated in the following analysis: Firstly, we assume that the RR sequence, *x(t),* is stationary (i.e., not time-correlated) normally distributed series. The assumption of non-stationarity may only hold over a short time period. Nevertheless, we can derive an expression for the correlation of the standard Lorenz coordinates as follows: Since the RR time sequence is normally distributed, the Lorenz plot coordinates, *X* and *Y*, are normal *difference* distributions*,* i.e. *[x(t)-x(t-1)]* and *[x(t-1)-x(t-2)]* are also normally distributed, but in this case with a mean and standard deviation of:

$\mu=0$ (1) $\sigma=\sqrt{2}\sigma_{R}$ (2)

The Pearson correlation coefficient between the two Lorenz coordinate series (i.e. *X* and *Y*) is calculated using the standard formula:

$\rho=\frac{Cov\left( X,Y \right)}{\sigma_{X}\sigma_{Y}}$ (3)

This can also be written as:

$\rho=\frac{E\left( XY \right)}{2{\sum X^{2}}/\left( n-1 \right)}$ (4)

Which, in expanded form, results in:

$\rho=\frac{E[x(t)-x(t-1)] [x(t-1)-x(t-2)]}{2{\sum X^{2}}/\left( n-1 \right)}$ (5)

Since *x(t), x(t-1)* and *x(t-2)* are uncorrelated (assumption of stationarity), the expectations, *E,* of their cross products are equal to zero. Therefore, the correlation coefficient can be simplified to:

$\rho=\frac{-E{[x(t-1)]}^{2}}{2{\sum X^{2}}/\left( n-1 \right)}$ (6)

Which further reduces to:

$\rho=\frac{{-\sum X^{2}}/\left( n-1 \right)}{{2\sum X^{2}}/\left( n-1 \right)}=-0.5$ (7)

$Type equation here.$Thus, the standard Lorenz plot coordinates are bivariate Normal with a correlation coefficient of -0.5. It is well known that in order to generate a correlated bivariate Gaussian (2D Normal) sequence, *X* and *Y* (in our case, the standard Lorenz plot coordinates) from 1D independent (i.e. uncorrelated) Gaussian sequences, *X* and *Y’*, then simply create a sequence as follows:

$Y=\rho X+Y'\sqrt{1-\rho^{2}}$ (8)

This is the inherent form of the *Y-*ordinate in the original correlated, i.e. standard Lorenz plot. In order to de-correlate this *Y*-ordinate, then simply invert the above equation to give:

$$Y'=\frac{Y-\rho X}{\sqrt{1-\rho^{2}}}$$

 (9)

Substituting for the Lorenz plot correlation coefficient derived earlier (i.e. -0.5) gives:

$Y'=\frac{Y+0.5X}{\sqrt{0.75}}$ (10)

If a Lorenz plot is now drawn with coordinates (*X, Y’*), instead of the original standard coordinates (*X, Y*), this will generate an uncorrelated bivariate normal distribution i.e. an uncorrelated Lorenz plot. In this way, any well-behaved RR time sequence will present as a symmetrical full quadrant pattern. An illustration of the decorrelation effect is shown below for a normal random RR interval sequence before (standard Lorenz) and after the transformation (decorrelated Lorenz).

A] Standard Lorenz Plot B] De-correlated Lorenz Plot

ms

ms

ms

ms

**Fig 1 – Standard and De-correlated Lorenz Plot for a normal random RR interval sequence**
